# Supplementary material for: Structural and energetic profiling of SARS-CoV-2 receptor binding domain antibody recognition and the impact of circulating variants
Source: PLoS Comput Biol. 2021 Sep 7;17(9):e1009380. doi: 10.1371/journal.pcbi.1009380 (PMC8448325; doi:10.1371/journal.pcbi.1009380)
Supplement: S1 Table — (DOCX) [file pcbi.1009380.s001.docx]

**S1 Table**. Antibody-spike and antibody-RBD complex structures analyzed in this study.

| **Antibody name** | **PDB code** | **Type^1^** | **Species^2^** | **IGHV gene^2^** | **Neut^3^** | **Resolution (Å)^4^** | **Structure Method^4^** | **Release Date^4^** |
| --- | --- | --- | --- | --- | --- | --- | --- | --- |
| Ab2-4 | 6XEY | ab | human | IGHV1-2 | Y | 3.25 | Cryo-EM | 7/21/20 |
| BD23 | 7BYR | ab | human | IGHV7-4-1 | Y | 3.84 | Cryo-EM | 6/9/20 |
| B38 | 7BZ5 | ab | human | IGHV3-66 | Y | 1.84 | X-ray | 5/12/20 |
| BD-236 | 7CHB | ab | human | IGHV3-53 | Y | 2.4 | X-ray | 9/15/20 |
| BD-368-2 | 7CHF | ab | human | IGHV3-23 | Y | 2.67 | X-ray | 9/15/20 |
| BD-604 | 7CHF | ab | human | IGHV3-53 | Y | 2.67 | X-ray | 9/15/20 |
| BD-629 | 7CH5 | ab | human | IGHV3-53 | Y | 2.7 | X-ray | 9/15/20 |
| C105 | 6XCM | ab | human | IGHV3-53 | Y | 3.42 | Cryo-EM | 6/30/20 |
| CB6 | 7C01 | ab | human | IGHV3-66 | Y | 2.88 | X-ray | 5/26/20 |
| CC12.1 | 6XC3 | ab | human | IGHV3-53 | Y | 2.7 | X-ray | 7/7/20 |
| CC12.3 | 6XC4 | ab | human | IGHV3-53 | Y | 2.34 | X-ray | 7/7/20 |
| COVA2-04 | 7JMO | ab | human | IGHV3-53 | Y | 2.36 | X-ray | 8/25/20 |
| COVA2-39 | 7JMP | ab | human | IGHV3-53 | Y [1] | 1.71 | X-ray | 8/25/20 |
| CR3022 | 6YLA | ab | human | IGHV5-51 | N | 2.42 | X-ray | 4/14/20 |
| CV30 | 6XE1 | ab | human | IGHV3-53 | Y | 2.75 | X-ray | 6/30/20 |
| EY6A | 6ZCZ | ab | human | IGHV3-30-3 | Y | 2.65 | X-ray | 6/23/20 |
| H014 | 7CAI | ab | human | IGHV1-69-2 | Cross | 3.49 | Cryo-EM | 9/22/20 |
| H11-D4 | 6YZ5 | nano | llama | IGHV3-3 | Y | 1.8 | X-ray | 6/2/20 |
| H11-H4 | 6ZH9 | nano | llama | IGHV3-3 | Y | 3.31 | X-ray | 9/1/20 |
| MR17 | 7C8W | nano | alpaca | IGHV3S53 | Y | 2.77 | X-ray | 6/23/20 |
| P2B-2F6 | 7BWJ | ab | human | IGHV4-38-2 | Y | 2.85 | X-ray | 6/2/20 |
| REGN10933 | 6XDG | ab | human | IGHV3-11 | Y | 3.9 | Cryo-EM | 6/23/20 |
| REGN10987 | 6XDG | ab | human | IGHV3-30 | Y | 3.9 | Cryo-EM | 6/23/20 |
| S309 | 7JX3 | ab | human | IGHV1-18 | Cross | 2.65 | X-ray | 10/14/20 |
| SR4 | 7C8V | nano | alpaca | IGHV3-3 | Y | 2.15 | X-ray | 6/23/20 |
| Ty1 | 6ZXN | nano | alpaca | IGHV3-48 | Y | 2.93 | Cryo-EM | 9/22/20 |
| S2M11 | 7K43 | ab | human | IGHV1-58 | Y | 2.6 | Cryo-EM | 10/7/2020 |
| S2E12 | 7K4N | ab | human | IGHV1-2 | Y | 3.3 | Cryo-EM | 10/7/2020 |
| S2A4 | 7JVC | ab | human | IGHV3-7 | Y | 3.3 | Cryo-EM | 10/14/20 |
| S2H13 | 7JV6 | ab | human | IGHV3-7 | Y | 3 | Cryo-EM | 10/14/20 |
| COVA1-16 | 7JMW | ab | human | IGHV1-46 | Cross | 2.89 | X-ray | 10/14/20 |
| CV07-250 | 6XKQ | ab | human | IGHV1-18 | Y | 2.55 | X-ray | 10/14/20 |
| CV07-270 | 6XKP | ab | human | IGHV3-11 | Y | 2.72 | X-ray | 10/14/20 |
| S304 | 7JX3 | ab | human | IGHV3-13 | Cross | 2.65 | X-ray | 10/14/20 |
| S2H14 | 7JX3 | ab | human | IGHV3-15 | Y | 2.65 | X-ray | 10/14/20 |
| C144 | 7K90 | ab | human | IGHV3-53 | Y | 3.24 | Cryo-EM | 10/21/20 |
| C135 | 7K8Z | ab | human | IGHV3-30 | Y | 3.5 | Cryo-EM | 10/21/20 |
| C121 | 7K8X | ab | human | IGHV1-2 | Y | 3.9 | Cryo-EM | 10/21/20 |
| C119 | 7K8W | ab | human | IGHV1-46 | Y | 3.6 | Cryo-EM | 10/21/20 |
| C110 | 7K8V | ab | human | IGHV5-51 | Y | 3.8 | Cryo-EM | 10/21/20 |
| C002 | 7K8T | ab | human | IGHV3-30 | Y | 3.4 | Cryo-EM | 10/21/20 |
| C102 | 7K8M | ab | human | IGHV3-53 | Y | 3.2 | X-ray | 10/21/20 |
| Sb23 | 7A29 | nano | alpaca | IGHV3-3 | Y | 2.94 | Cryo-EM | 10/21/20 |
| C104 | 7K8U | ab | human | IGHV4-34 | Y | 3.8 | Cryo-EM | 10/21/20 |
| 298 | 7K9Z | ab | human | IGHV1-2 | Y | 2.95 | X-ray | 10/28/20 |
| 52 | 7K9Z | ab | human | IGHV1-69 | Y | 2.95 | X-ray | 10/28/20 |
| mNb6 | 7KKL | nano | alpaca | IGHV3S53 | Y | 2.85 | Cryo-EM | 11/11/20 |
| P2C-1A3 | 7CDJ | ab | human | IGHV3-11 | Y | 3.4 | X-ray | 11/18/20 |
| P2C-1F11 | 7CDI | ab | human | IGHV3-66 | Y | 2.96 | X-ray | 11/18/20 |
| P4A1 | 7CJF | ab | human | IGHV3-53 | Y | 2.11 | X-ray | 11/11/2020 |
| C1A-B12 | 7KFV | ab | human | IGHV3-53 | Y | 2.1 | X-ray | 12/2/2020 |
| Nb20 | 7JVB | nano | alpaca | IGHV3-3 | Y | 3.29 | X-ray | 12/2/2020 |
| 2H2 | 7DK4 | ab | mouse | IGHV2-5-1 | Y | 3.8 | Cryo-EM | 12/2/2020 |
| P17 | 7CWN | ab | human | IGHV1-69-2 | Y | 3.2 | Cryo-EM | 12/16/2020 |
| STE90-C11 | 7B3O | ab | human | IGHV3-66 | Y | 2 | X-ray | 12/16/2020 |
| CR3014-C8 | 7KZB | ab | human | IGHV3-72 | N | 2.83 | X-ray | 2/3/2021 |
| Sb16 | 7KGK | nano | alpaca | IGHV3S53 | Y | 2.6 | X-ray | 2/3/2021 |
| Sb45 | 7KGJ | nano | alpaca | IGHV3S53 | N | 2.3 | X-ray | 2/3/2021 |
| DH1047 | 7LD1 | ab | human | IGHV1-46 | Y | 3.4 | Cryo-EM | 1/27/2021 |
| LY-CoV481 | 7KMI | ab | human | IGHV3-53 | Y | 1.73 | X-ray | 1/27/2021 |
| LY-CoV488 | 7KMH | ab | human | IGHV3-53 | Y | 1.72 | X-ray | 1/27/2021 |
| LY-CoV555 | 7KMG | ab | human | IGHV1-69 | Y | 2.16 | X-ray | 1/27/2021 |
| W | 7KN7 | nano | alpaca | IGHV3-3 | Y | 2.73 | X-ray | 1/20/2021 |
| V | 7KN6 | nano | alpaca | IGHV3S1 | Y | 2.55 | X-ray | 1/20/2021 |
| E | 7KN5 | nano | alpaca | IGHV3-3 | Y | 1.87 | X-ray | 1/20/2021 |
| U | 7KN5 | nano | alpaca | IGHV3-3 | Y | 1.87 | X-ray | 1/20/2021 |
| CT-P59 | 7CM4 | ab | human | IGHV2-70 | Y [2] | 2.71 | X-ray | 1/20/2021 |
| 2-15 | 7L5B | ab | human | IGHV1-2 | Y | 3.18 | X-ray | 2/10/2021 |
| 15033-7 | 7KLH | ab | human | IGHV3-23 | Y | 3 | X-ray | 2/10/2021 |
| Sb68 | 7KLW | nano | alpaca | IGHV3S53 | Y | 2.6 | X-ray | 2/3/2021 |

^1^Antibody type. “ab”: heavy-light chain antibody, “nano”: nanobody/VHH.

^2^Species and IGHV gene name determined by ANARCI from antibody heavy chain or nanobody sequence.

^3^Measured SARS-CoV-2 neutralization, from CoV-AbDab [3] or the literature, where specified by a reference. N: does not neutralize SARS-CoV-2; Y: neutralizes SARS-CoV-2; Cross: neutralizes SARS-CoV-2 and SARS-CoV-1.

^4^Resolution, structure determination method, and release date of structure in the Protein Data Bank (PDB) [4]. Cryo-EM: cryogenic electron microscopy, X-ray: X-ray diffraction.

**References**

1. Brouwer PJM, Caniels TG, van der Straten K, Snitselaar JL, Aldon Y, Bangaru S, et al. Potent neutralizing antibodies from COVID-19 patients define multiple targets of vulnerability. Science. 2020;369(6504):643-50. Epub 2020/06/17. doi: 10.1126/science.abc5902. PubMed PMID: 32540902; PubMed Central PMCID: PMC7299281.

2. Kim C, Ryu DK, Lee J, Kim YI, Seo JM, Kim YG, et al. A therapeutic neutralizing antibody targeting receptor binding domain of SARS-CoV-2 spike protein. Nat Commun. 2021;12(1):288. Epub 2021/01/14. doi: 10.1038/s41467-020-20602-5. PubMed PMID: 33436577; PubMed Central PMCID: PMC7803729.

3. Raybould MIJ, Kovaltsuk A, Marks C, Deane CM. CoV-AbDab: the Coronavirus Antibody Database. Bioinformatics. 2020. Epub 2020/08/18. doi: 10.1093/bioinformatics/btaa739. PubMed PMID: 32805021; PubMed Central PMCID: PMC7558925.

4. Rose PW, Beran B, Bi C, Bluhm WF, Dimitropoulos D, Goodsell DS, et al. The RCSB Protein Data Bank: redesigned web site and web services. Nucleic Acids Research. 2011;39(Database issue):D392-401. Epub 2010/11/03. doi: 10.1093/nar/gkq1021. PubMed PMID: 21036868; PubMed Central PMCID: PMC3013649.
