# Supplementary material for: Structural and energetic profiling of SARS-CoV-2 receptor binding domain antibody recognition and the impact of circulating variants
Source: PLoS Comput Biol. 2021 Sep 7;17(9):e1009380. doi: 10.1371/journal.pcbi.1009380 (PMC8448325; doi:10.1371/journal.pcbi.1009380)
Supplement: S2 Table — (DOCX) [file pcbi.1009380.s002.docx]

**S2 Table**. Performance of computational alanine scanning ΔΔG prediction for antibody-antigen interfaces.

| **Method** | **Correlation^1^** | **AUC^2^** |
| --- | --- | --- |
| FoldX | 0.49 | 0.67 |
| Ros2.3_norepack | 0.52 | 0.71 |
| Ros2.3_minint_bb_chi | 0.50 | 0.70 |
| **Ros2.3_minint_chi** | **0.53** | **0.72** |
| Ros2.3_repack | 0.51 | 0.70 |
| Ros3_norepack | 0.44 | 0.68 |
| Ros3_repack | 0.45 | 0.69 |
| flex_ddG-fa_talaris2014 | 0.48 | 0.66 |
| flex_ddG-fa_talaris2014-gam | 0.52 | 0.69 |
|  |  |  |
| *Including non-alanine mutants* | | |
| Ros2.3_minint_chi^3^ | 0.50 | 0.70 |

Computational predictions of binding affinity changes were computed for a subset of the AB-Bind dataset of measured antibody-antigen ΔΔG values [1] with alanine point substitutions, available wild-type complex structures, and quantified ΔΔG measurements (347 total ΔΔGs). Rosetta version 2.3 (Ros2.3) [2], Rosetta version 3.12 (Ros3) [3], and FoldX version 4 [4] were used to predict ΔΔG values using different modeling and scoring protocols, as detailed in the Methods. Protocol selected for RBD alanine scanning based on performance comparison is shown in **bold**.

^1^Pearson correlation coefficient between predicted and experimentally determined ΔΔG values.

^2^ROC AUC value for prediction of hotspot (experimental ΔΔG > 1 kcal/mol) versus non-hotspot residues based on predicted ΔΔG values.

^3^Predictive performance for larger AB-Bind set that includes non-alanine point substitutions (531 mutants and ΔΔG values).

**References**

1. Sirin S, Apgar JR, Bennett EM, Keating AE. AB-Bind: Antibody binding mutational database for computational affinity predictions. Protein Sci. 2016;25(2):393-409. doi: 10.1002/pro.2829. PubMed PMID: 26473627; PubMed Central PMCID: PMC4815335.

2. Kortemme T, Kim DE, Baker D. Computational alanine scanning of protein-protein interfaces. Sci STKE. 2004;2004(219):pl2. PubMed PMID: 14872095.

3. Leman JK, Weitzner BD, Lewis SM, Adolf-Bryfogle J, Alam N, Alford RF, et al. Macromolecular modeling and design in Rosetta: recent methods and frameworks. Nat Methods. 2020;17(7):665-80. Epub 2020/06/03. doi: 10.1038/s41592-020-0848-2. PubMed PMID: 32483333; PubMed Central PMCID: PMC7603796.

4. Schymkowitz J, Borg J, Stricher F, Nys R, Rousseau F, Serrano L. The FoldX web server: an online force field. Nucleic Acids Res. 2005;33(Web Server issue):W382-8. Epub 2005/06/28. doi: 10.1093/nar/gki387. PubMed PMID: 15980494; PubMed Central PMCID: PMC1160148.
