# Supplementary material for: Structural and energetic profiling of SARS-CoV-2 receptor binding domain antibody recognition and the impact of circulating variants
Source: PLoS Comput Biol. 2021 Sep 7;17(9):e1009380. doi: 10.1371/journal.pcbi.1009380 (PMC8448325; doi:10.1371/journal.pcbi.1009380)
Supplement: S3 Table — (DOCX) [file pcbi.1009380.s003.docx]

**S3 Table**. Comparison of ΔΔG predictions with measured monoclonal antibody neutralization of SARS-CoV-2 variants from Wang et al. [1].

|  | **N501Y** | | | **K417N** | | | **E484K** | | | **Alpha** | | | **Beta** | | |
| --- | --- | --- | --- | --- | --- | --- | --- | --- | --- | --- | --- | --- | --- | --- | --- |
| **Antibody** | **Exp^1^** | **Ros^2^** | **FoldX^3^** | **Exp^1^** | **Ros^2^** | **FoldX^3^** | **Exp^1^** | **Ros^2^** | **FoldX^3^** | **Exp^1^** | **Ros^2^** | **FoldX^3^** | **Exp^1^** | **Ros^2^** | **FoldX^3^** |
| 2-15 | 1.5 | 0.0 | 0.1 | 3.3 | 0.0 | -0.1 | N | 0.6 | 0.2 | -3 | 0.0 | 0.1 | N | 0.6 | -0.5 |
| LY-CoV555 | -1 | 0.0 | 0.0 | 8.4 | 0.1 | -0.1 | N | 2.9 | 12.7 | -2.8 | 0.0 | 0.0 | N | 2.9 | 12.5 |
| REGN10933 | -1.4 | -0.1 | -0.6 | -13.1 | 0.1 | 1.0 | -10.5 | 1.6 | 1.2 | 1 | -0.1 | -0.6 | N | 1.6 | 1.8 |
| C121 | 1.5 | 0.0 | 0.1 | 1.2 | 0.0 | -0.1 | N | 0.0 | -0.2 | 4 | 0.0 | 0.1 | N | 0.1 | 0.1 |
| REGN10987 | 1.3 | 0.0 | 0.1 | -1.2 | 0.0 | 0.0 | -1.1 | 0.0 | 0.0 | 1 | 0.0 | 0.1 | -3.5 | 0.0 | 0.1 |
| S309 | 1.2 | 0.0 | 0.0 | 1.6 | 0.0 | 0.0 | 2.5 | 0.0 | 0.0 | -4 | 0.0 | 0.0 | -2.2 | 0.0 | 0.0 |
| COVA1-16 | -1.4 | 0.0 | 0.0 | 3.3 | 0.0 | 0.0 | -1 | 0.0 | 0.0 | 3.4 | 0.0 | 0.0 | 1 | 0.0 | 0.0 |

^1^Experimentally determined neutralization of viral variant, from Wang et al. [1] (Fig 2a in that study). Values reflect fold change in antibody neutralization (IC_50_) for variant versus wild-type virus, with negative values indicating lower neutralization (higher IC_50_). “N” indicates unquantifiable neutralization (< -1000) in Wang et al.. Measurements of greater that 10-fold loss of neutralization (< -10, or “N”) are highlighted by red cells.

^2^Rosetta ΔΔG for viral variant based on mutagenesis of RBD, in Rosetta Energy Units (REU) which are comparable to energies in kcal/mol. Predicted disruptive effects (ΔΔG > 1.0) have cells shaded red.

^3^FoldX ΔΔG for viral variant based on mutagenesis of RBD, in units of kcal/mol. Predicted disruptive effects (ΔΔG > 1.0) have cells shaded red.

**References**

1. Wang P, Nair MS, Liu L, Iketani S, Luo Y, Guo Y, et al. Antibody resistance of SARS-CoV-2 variants B.1.351 and B.1.1.7. Nature. 2021;593(7857):130-5. Epub 2021/03/09. doi: 10.1038/s41586-021-03398-2. PubMed PMID: 33684923.
