# Supplementary material for: Structural and energetic profiling of SARS-CoV-2 receptor binding domain antibody recognition and the impact of circulating variants
Source: PLoS Comput Biol. 2021 Sep 7;17(9):e1009380. doi: 10.1371/journal.pcbi.1009380 (PMC8448325; doi:10.1371/journal.pcbi.1009380)
Supplement: S4 Table — (DOCX) [file pcbi.1009380.s004.docx]

**S4 Table**. Comparison of ΔΔG predictions with measured monoclonal antibody neutralization of SARS-CoV-2 variants from Planas et al. [1].

|  | **Alpha** | | | **Beta** | | | **Delta** | | | |
| --- | --- | --- | --- | --- | --- | --- | --- | --- | --- | --- |
| **Antibody** | **Exp^1^** | **Ros^2^** | **FoldX^3^** | **Exp^1^** | **Ros^2^** | **FoldX^3^** | **Exp^1^** | **Ros^2^** | **FoldX^3^** |  |
| LY-CoV555 | Y | 0 | 0 | N | 2.9 | 12.5 | N | -0.2 | 1.5 |  |
| REGN10933 | Y | -0.1 | -0.6 | N | 1.6 | 1.8 | Y | 0 | -0.8 |  |
| REGN10987 | Y | 0 | 0.1 | Y | 0.1 | 0.1 | Y | 0 | -0.2 |  |

^1^Experimentally determined neutralization of viral variant, from Planas et al. [1] (Fig 1 in that study). Y: antibody neutralization; N: low or no antibody neutralization (cells shaded red).

^2^Rosetta ΔΔG for viral variant based on mutagenesis of RBD, in Rosetta Energy Units (REU) which are comparable to energies in kcal/mol. Predicted disruptive effects (ΔΔG > 1.0) have cells shaded red.

^3^FoldX ΔΔG for viral variant based on mutagenesis of RBD, in units of kcal/mol. Predicted disruptive effects (ΔΔG > 1.0) have cells shaded red.

**References**

1. Planas D, Veyer D, Baidaliuk A, Staropoli I, Guivel-Benhassine F, Rajah MM, et al. Reduced sensitivity of SARS-CoV-2 variant Delta to antibody neutralization. Nature. 2021;596(7871):276-80. Epub 2021/07/09. doi: 10.1038/s41586-021-03777-9. PubMed PMID: 34237773.
