# Supplementary material for: Structural and energetic profiling of SARS-CoV-2 receptor binding domain antibody recognition and the impact of circulating variants
Source: PLoS Comput Biol. 2021 Sep 7;17(9):e1009380. doi: 10.1371/journal.pcbi.1009380 (PMC8448325; doi:10.1371/journal.pcbi.1009380)
Supplement: S1 Fig — (A) Resolution, (B) interface buried surface area (BSA), and (C) number of interface atomic contacts between antibody and RBD within a 5 Å distance cutoff were compared for structures obtained by cryo-EM and X-ray diffraction. Structures containing antibodies and nanobodies were separated to avoid possible bias in interface size due to smaller size of nanobodies. Statistical significance (Wilcoxon rank-sum test) between properties of cryo-EM and X-ray antibody-RBD structures is indicated at top (*: p < 0.05; **: p < 0.01; ***: p < 0.001). Due to small number of values for nanobody cryo-EM complex structures (N = 3), statistical comparisons were not performed for the nanobody-containing structures. (PDF) [file pcbi.1009380.s005.pdf]

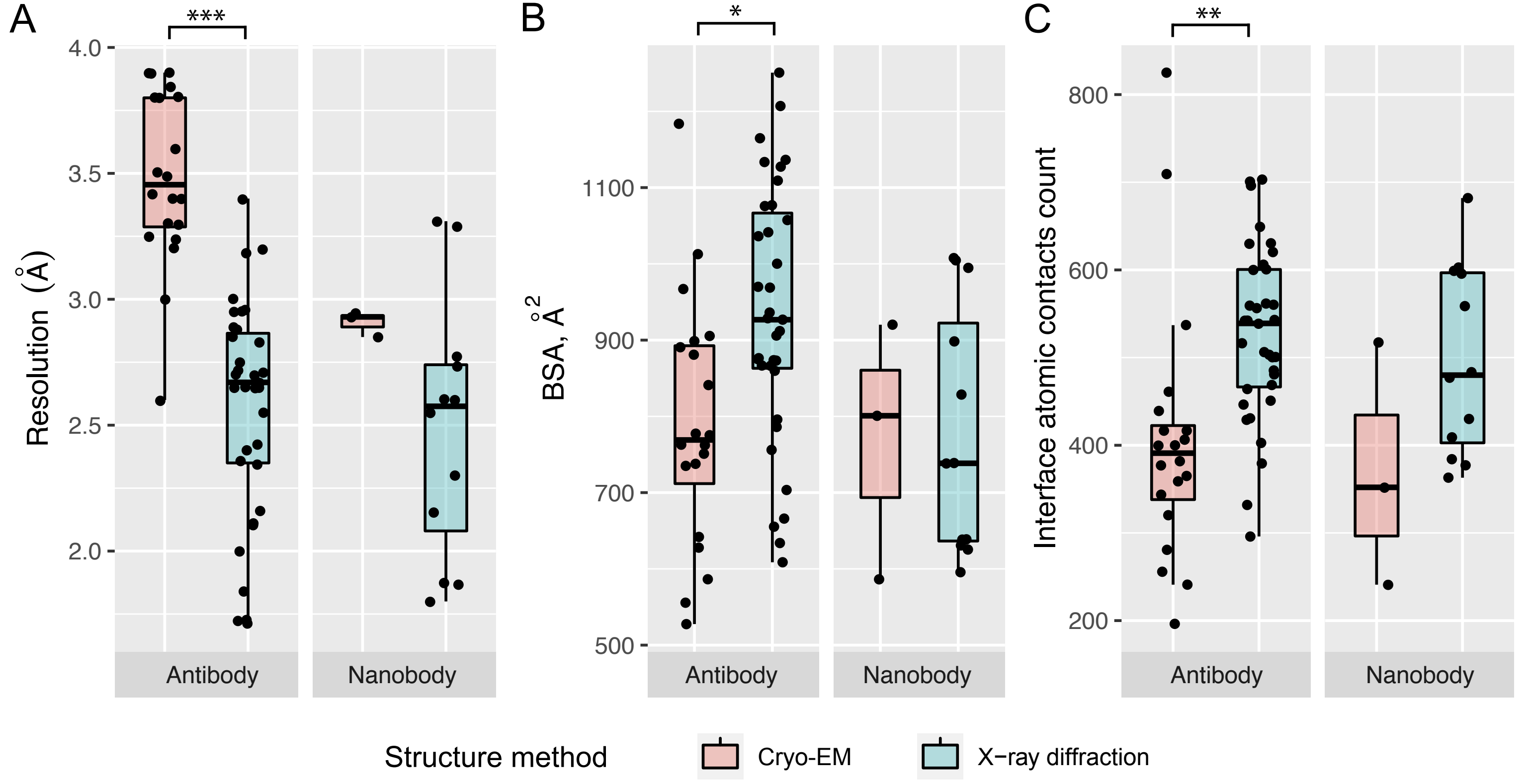
A
***
4.0
3.5
3.0
2.5
2.0
Resolution (Å)
Antibody
Nanobody
B
*
1100
900
700
500
BSA, Å2
Antibody
Nanobody
C
**
800
600
400
200
Interface atomic contacts count
Antibody
Nanobody
Structure method
Cryo-EM
X-ray diffraction
