# Supplementary figures and images for: Structural and energetic profiling of SARS-CoV-2 receptor binding domain antibody recognition and the impact of circulating variants
Source: PLoS Comput Biol. 2021 Sep 7;17(9):e1009380. doi: 10.1371/journal.pcbi.1009380 (PMC8448325; doi:10.1371/journal.pcbi.1009380)

**A**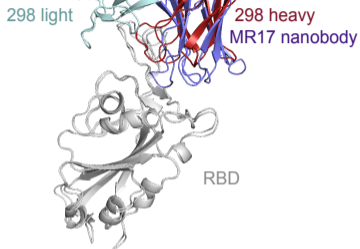**B**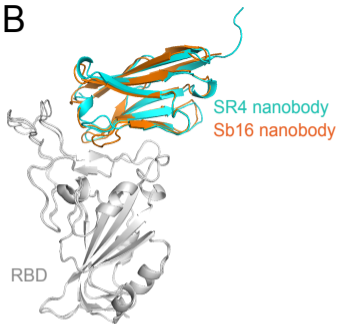**C**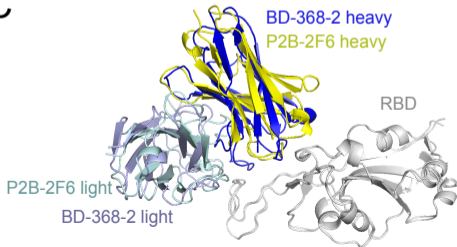

Supplement: S2 Fig — Shown are (A) antibodies MR17 (PDB code 7C8W) and 298 (PDB code 7K9Z), which have a 4.7 Å heavy chain orientation RMSD, (B) antibodies SR4 (PDB code 7C8V) and Sb16 (PDB code 7KGK), which have a 1.2 Å heavy chain orientation RMSD, and (C) antibodies BD-368-2 (PDB code 7CHF) and P2B-2F6 (PDB code 7BWJ), which have a 5.2 Å heavy chain orientation RMSD. The antibody-RBD structures are superposed by RBD (gray), and antibody chains (heavy, light, or nanobody) are colored separately as indicated. (PDF) [file pcbi.1009380.s006.pdf]

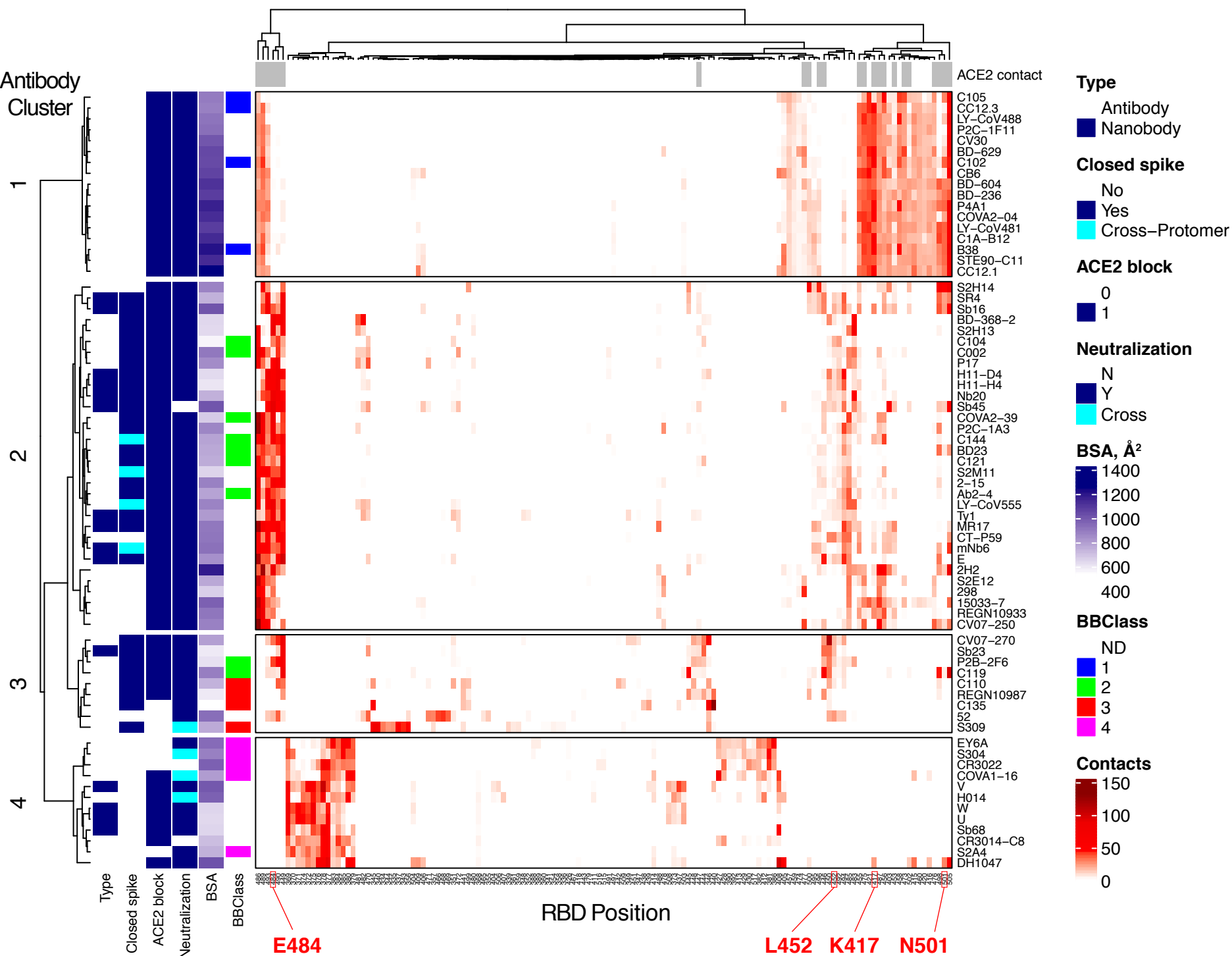

Supplement: S3 Fig — Labels and annotations are in accordance with the corresponding labels/annotations in Fig 2, and antibodies (rows) and RBD positions (columns) are ordered by hierarchical clustering in R. “BBClass” denotes the antibody classification from a previous study [23], with “ND” (empty cell) indicating that the class for the antibody was not described in that work. Antibodies in the heatmap are separated by the four major hierarchical clusters, which are labeled on left. (PDF) [file pcbi.1009380.s007.pdf]

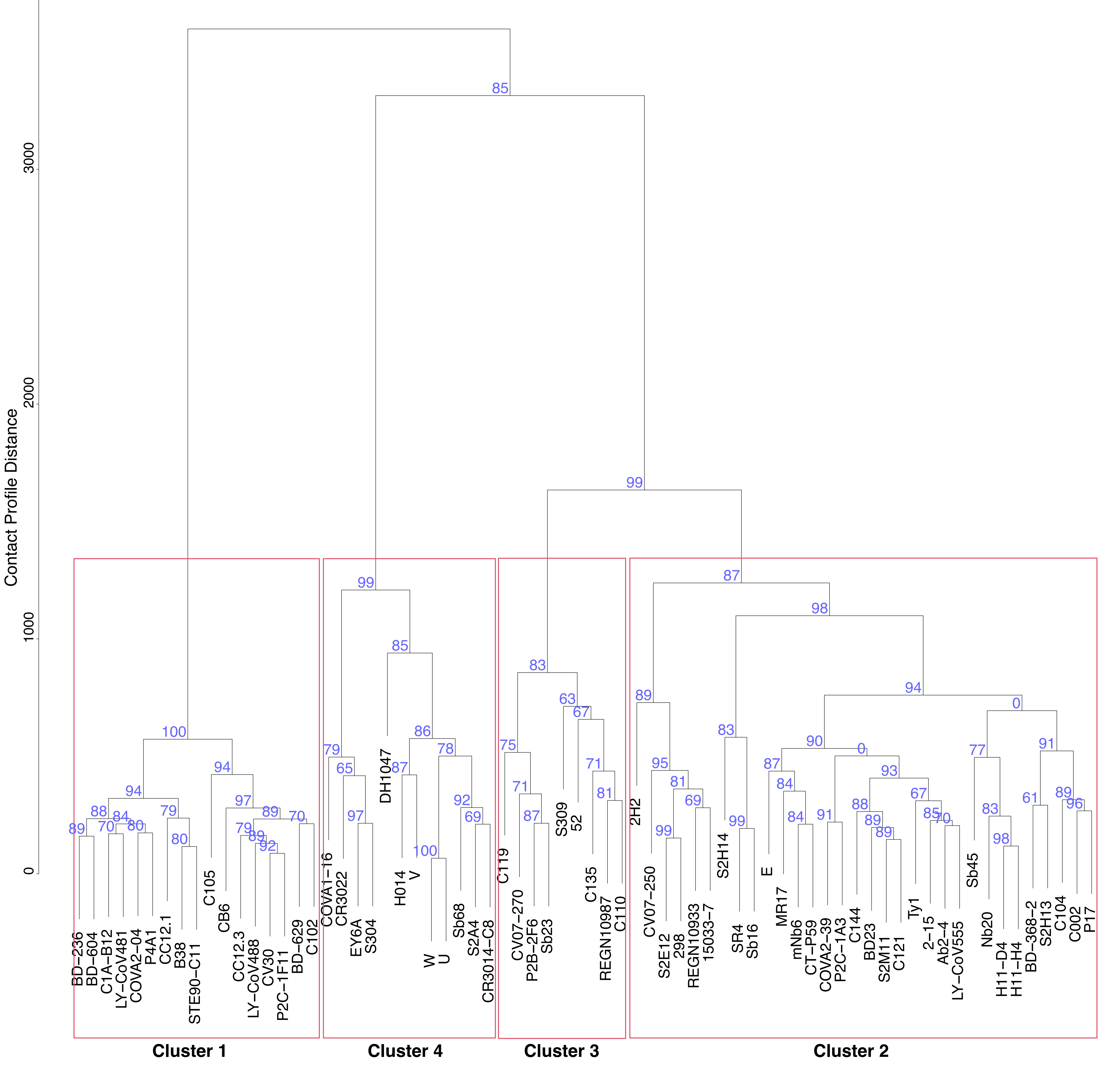

Supplement: S4 Fig — Multiscale bootstrap resampling was performed in pvclust [49] in R, with the antibody-RBD contact data and 10,000 replicates. Values at each node denote the Approximately Unbiased (AU) bootstrap confidence, and red boxes delineate the four major clusters noted in this study, labeled accordingly. (PDF) [file pcbi.1009380.s008.pdf]

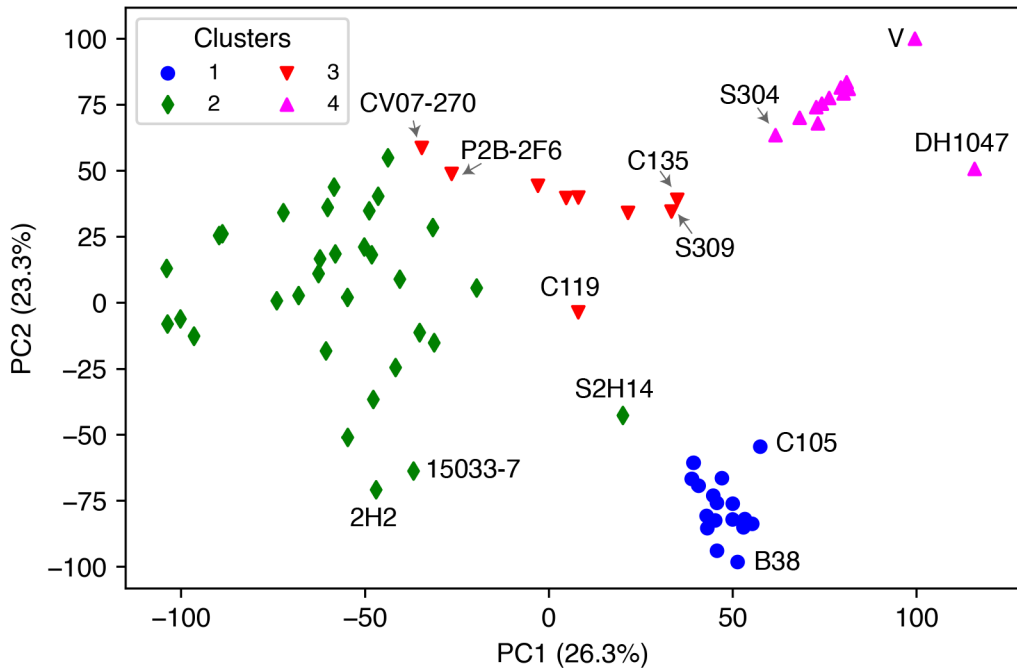

Supplement: S5 Fig — The x and y axes represent the first two principal components (PC1, PC2), with percentage of data variance represented by each principal component shown in parentheses. The 70 antibodies are shown as points, with colors and shapes representing Clusters 1–4, which were determined by hierarchical clustering analysis of antibody-RBD contact profiles. Selected points representing antibodies that are located on the periphery of cluster distributions are labeled by corresponding antibody names. (PDF) [file pcbi.1009380.s009.pdf]

K417N

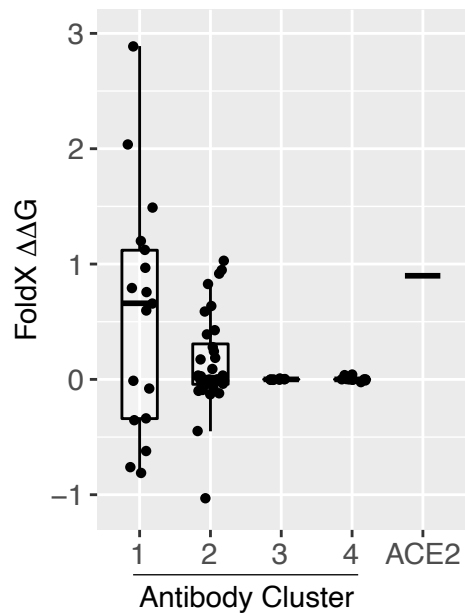

K417T

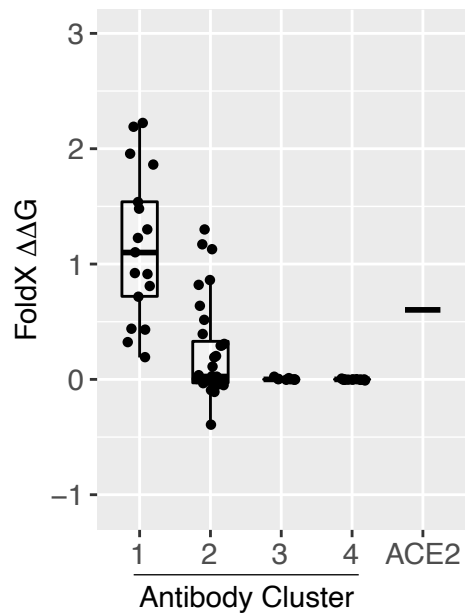

L452R

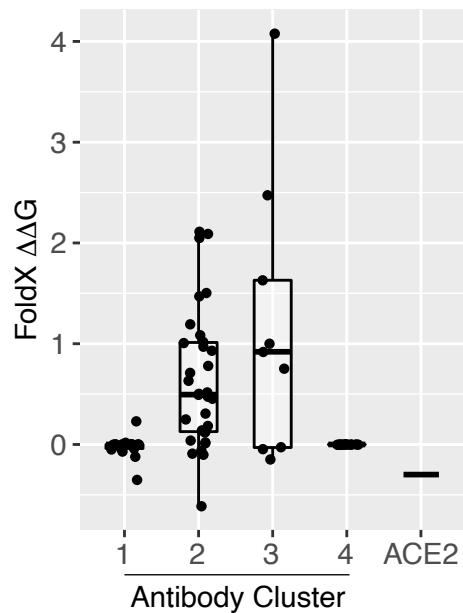

S477N

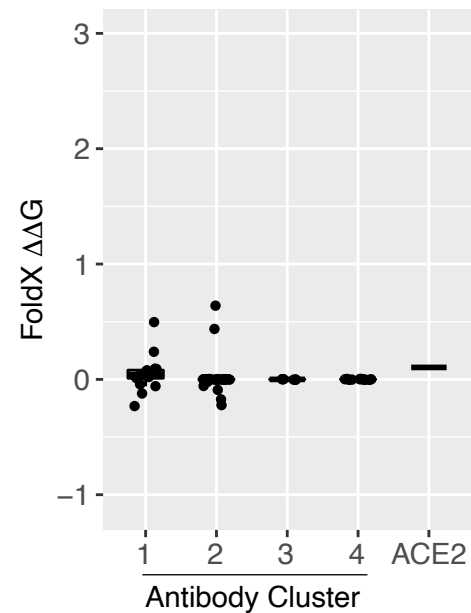

T478K

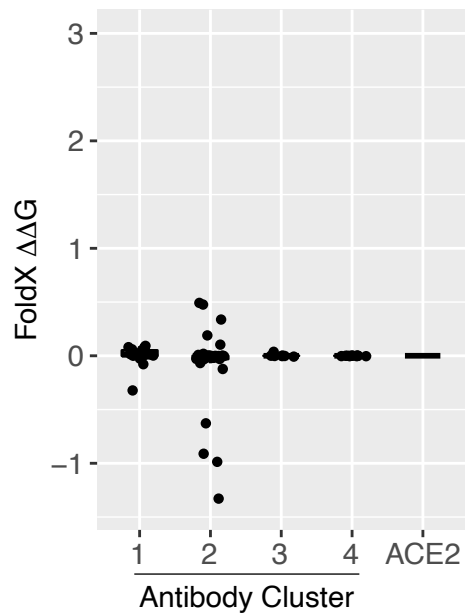

E484K

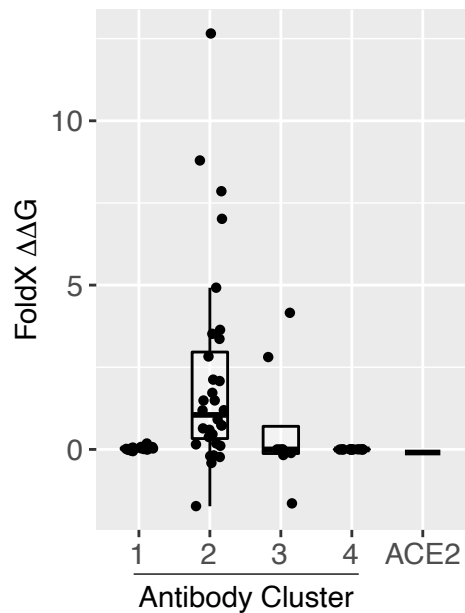

E484Q

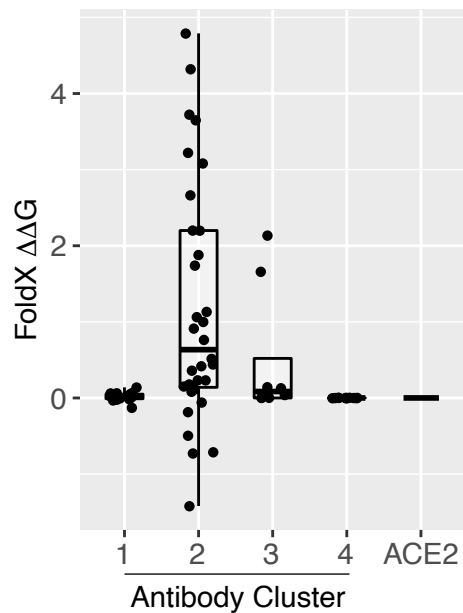

N501Y

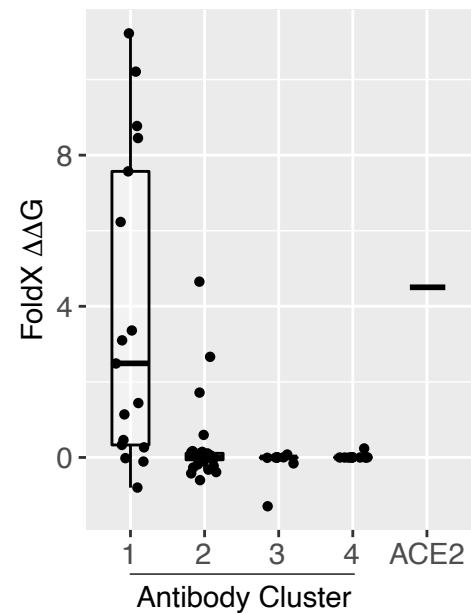

Supplement: S6 Fig — FoldX [57] was used to simulate and compute binding affinity changes (ΔΔGs, in units of kcal/mol) for RBD point substitutions in 70 antibody-RBD complex structures and the ACE2-RBD complex structure (PDB code 6LZG). ΔΔG values for each RBD substitution are shown as a separate boxplot, with antibodies grouped by contact based cluster (1–4). The ACE2 ΔΔG value for each RBD point substitution is shown as a horizontal bar. (PDF) [file pcbi.1009380.s010.pdf]
