# Supplementary material for: Structural and energetic profiling of SARS-CoV-2 receptor binding domain antibody recognition and the impact of circulating variants
Source: PLoS Comput Biol. 2021 Sep 7;17(9):e1009380. doi: 10.1371/journal.pcbi.1009380 (PMC8448325; doi:10.1371/journal.pcbi.1009380)
Supplement: Attachment — Submitted filename: response_to_reviewers.pdf [file pcbi.1009380.s011.pdf]

We are grateful to the reviewers for their time and for their helpful comments and suggestions. We have edited the manuscript to address all of the comments of the reviewers. In addition to edits of the figures, tables and text, we have added one new main figure, two new supplemental figures, and two new supplemental tables to respond to the reviewers' comments. These revisions enhance this study and provide further support for our conclusions. Responses to specific comments are below, with the original reviewers' comments in blue italics.

Reviewer #1:

In this manuscript, Yin et al. perform epitope interaction analysis across a set of 70 solved 3D antibody:SARS-CoV-2 RBD structures, with the aim of predicting the likely impact of circulating variants on antibody recognition. First antibody "footprints" were derived through antigen contact analysis, and four broad epitope clusters were found through unsupervised clustering of these footprints. Mutational alanine scanning was then performed on each complex to identify key attractive binding interactions, and the impact of the specific mutations present in some SARS-CoV-2 viral variants were also assessed to deduce whether they are likely to have arisen to evade immune protection.

Though the authors' approach for discretising SARS-CoV-2 epitope regions via. clustering antibody footprints is novel, by this stage in the pandemic the conclusions of this part of the work are largely confirmatory (see Barnes et al. [https://doi.org/10.1038/s41586-020-2852-1], Dejnirattisai et al. [https://doi.org/10.1016/j.cell.2021.02.032], and Niu et al. [https://doi.org/10.3389/fimmu.2021.647934]). The more impactful aspect of this study is that the authors proceeded to study the conservation of binding interactions within these epitope clusters, establishing a computational framework by which the energetic impact of viral variants can be predicted for the antibodies within. Effective computational workflows for this purpose will be important to rapidly highlight novel variants as "of concern" and to inform future vaccination strategies.
The paper is well-constructed, and an excellent level of detail is supplied in the methods to ensure reproducibility. I have a few minor concerns/suggestions for alterations to the paper:

1. The manuscript should be clearer that both X-ray crystal structures and cryo-EM structures are being analysed side-by-side in this dataset of 70 structures (perhaps in the main text and as an additional column to Table S1).

In my experience, cryo-EM structures tend to offer good information about approximate antibody binding region but can understate the number of interactions holding the complex together as side-chain information is often not well resolved. Were the cryo-EM structures on average of lower resolution? Was "score" required more often to fill-in side chains for the cryo-EM structures than for the XRD structures? I would be interested to see some summative statistics across the set of cryo-EM and XRD structures (perhaps split by antibody/nanobody) to capture whether fewer contacts/interactions or lower binding site interaction energies tend to be found in cryo-EM structures. If this is the case, please could the authors comment on whether the resulting sparsity of recorded interactions has led/could lead to uncertainty or errors in binding

cluster assignment, particularly at the periphery of region definitions?

In response to this comment, we have added a new supplemental figure (Figure S1) that compares resolutions, interface surface area, and number of contacts for X-ray versus cryo-EM structures in this set. We have also included annotation of structure method (X-ray, cryo-EM) in Table S1. Indeed, as the reviewer suggested, there are some differences between the X-ray and cryo-EM structures. This is noted in the main text (lines 79-83):

“As noted in **Table S1**, all structures were obtained by X-ray diffraction or cryogenic electron microscopy (cryo-EM), and while the cryo-EM structures had significantly lower resolutions ($p < 0.001$), as expected, antibody-RBD interface size and number of inter-molecular atomic contacts were also somewhat lower for cryo-EM structures, albeit with less significance.”

Also, we have added a note regarding cluster confidence and structure quality, in accordance with the comment of the reviewer (lines 119-122):

“Due to the moderately lower bootstrap confidence, it is possible that some antibodies from Clusters 2 and 3, particularly those proximal to the inter-cluster boundaries and including some cryo-EM structures that have poorer resolutions (**Figure S1**), could have potential ambiguity in Cluster 2 versus Cluster 3 assignments.”

2. I think figure 5 would be augmented with a profile of ΔΔG of each RBD position for the binding of ACE-2 to the RBD (just as the authors did for figure 2). This goes also for figure 7/S4/S5, where the predicted ΔΔG impact of each mutation on ACE-2 binding would provide a relevant reference point to determine whether the mutations might have dual purpose for improving ACE-2 binding while escaping neutralising antibodies, improve ACE-2 binding while not escaping neutralising antibodies, or might even be deleterious to ACE-2 binding but even so be accommodated due to the benefit of immune evasion.

We agree with this comment from the reviewer, and have now included ACE2 ΔΔG information in Figure 5, Figure 7, Figure 8, Figure S6, as well as ACE2-RBD hydrogen bond information in Figure 4. We have added a note regarding the predicted and experimentally measured impact of variants on ACE2 binding in the Results (lines 211-216).

3. Relatedly, a timely addition to the paper would be analysis of the new "Indian" variant of concern (B1.617). What is the predicted impact of E484Q on cluster 2 antibodies, compared with E484K? And on ACE-2 binding? L452R, which was previously in the SI, could be brought into the main manuscript in accordance with its rising profile as a feature of the B1.617 variant. The authors could also consider analysing the combined affect on ΔΔG of multiple mutations (i.e. E484Q+L452R) in addition to analysing the mutations separately.

We appreciate this suggestion from the reviewer, and have now included that variant, which is referred to in the manuscript as Delta (per the recent convention), both as individual substitutions in Figure 7 (L452R, T478K) and as combined substitutions in Figure 8, along with

other variants of concern (Alpha, Beta, Gamma). We have also included E484Q in Figure 7, and have added effects on ACE2 binding for the individual substitutions in Figure 7 and the combined substitutions in Figure 8. The impact of the Delta variant is also the focus of a new paragraph in the Discussion (pgs. 259-268).

4. As the study is limited to analysis of antibodies against the receptor binding domain, I think the title should read "...SARS-CoV-2 receptor-binding domain antibody recognition...". Many studies are now showing antibodies can engage SARS-CoV-2 in many other regions.

We have updated the title according to the reviewer's suggestion.

Typos
Line 118: I think this should be a to reference Figure S2 instead of S1.
Line 120: "Visualization the"
Line 135: "most are predicted block ACE2"
Line 175: "lusters"
Line 311: "which identifies and antibody-RBD structures"

These typos have been corrected. Figure S2 is now Figure S4, so the corrected reference in the text is to Figure S4.

Reviewer #2:
It is vitally important that we understand the molecular interactions that underpin SARS-CoV-2 antibody-mediated neutralisation, and predict the pathways by which SARS-CoV-2 may escape immunity. In this study, Yin et. al. draw upon the wealth of Spike RBD-antibody structures to provide an integrated perspective on SARS-CoV-2 neutralisation. This work has two major values. First, through functional/structural classification the authors identify 4 clusters of anti-RBD antibodies; this provides a framework to understand and evaluate the ever-increasing list of anti-Spike mAbs/nanobodies. Secondly, by examining the theoretical resilience of antibody binding to mutation, the authors investigate the impact of on going, and future, antigenic variation. They also identify a particular cluster of antibodies that may withstand mutational escape and, therefore, would be a desirable specificity in vaccine-induced immunity. This work is of immediate value and importance, providing clarity on Spike-antibody interactions. I support the publication of this manuscript, however, there are some revisions that, in my opinion, would significantly improve the study.

Major Comments
• The categorisation and analysis of RBD-mAb interactions allow the authors to make predictions on the antigenic escape of certain mAbs, particularly in the context of recently emergent SARS-CoV-2 variants. It would be desirable to experimentally test a few of those predictions, this could be achieved with pseudovirus and synthesised antibodies. However, it may also be sufficient to verify predictions using analogous datasets in publications from others.

In response to this comment, we have added two supplemental Tables (Tables S3 and S4) comparing predicted ΔΔG values with experimentally measured neutralization values for a number of monoclonal antibodies, and variants including Alpha, Beta, and Delta. We found that the predictions largely match experimental neutralization measurements, and we opted to include FoldX in Figure 8, in addition to Rosetta, based on its predictive performance in Tables S3 and S4. The associated text describing these new results is in lines 225-232. We have also included a note in the Results regarding the predicted versus experimental impact on ACE2 affinities for certain RBD substitutions (lines 211-216).

• *A great value of this work is the analytical summary and organisation of a lot of important information regarding spike mAbs. Consequently the reader can gain a broad perspective of spike-antibody interactions. However, I think the authors are underselling their work by not communicating clearly. This is particularly the case in the figures where there is inadequate description or depiction of their findings. I provide some suggestions below.*

We appreciate these suggestions from the reviewer, and have responded to the specific comments below.

• *The discussion has a little too much focus on what could be done in the future to expand on this work. This somewhat detracts from the intrinsic value of the existent work presented in the manuscript. The discussion should be rebalanced to included more interpretation and contextualisation of the study alongside the literature.*

We agree with the reviewer on this comment, and the Discussion section has been revised accordingly. The Discussion has been modified to remove much of the discussion on future work, including the paragraph on the inclusion of ACE2 effects which is now obsolete. This we believe has improved the balance of the Discussion. Additionally, we have included new text and references focused on interpretation and contextualization, including lines 260-269 regarding the Delta variant, and lines 313-315 regarding recently described cross-reactive monoclonal antibodies.

Figure revision suggestions:
Figure 1: Maybe a few more representative superpositions of antibodies (in the supplementary material?). Also the dendogram is hard to read (e.g. the dashed line is not clear), can the branch lengths be expressed on a log scale to reveal the finer details of clustering? Also, where example structures are given highlight them on the dendogram to aid comprehension.

We have added a new supplemental figure (Figure S2) that shows three more pairs of representative superposed antibody structures, and have also modified Figure 1 to change the dendrogram to log scale and darken the dashed line. A dashed blue line has been added to delineate the location of the structures on the dendrogam that are shown in Figure 1B.

Figure 2: Is there value in including the BBClass? I appreciate it is relevant, but could also be

covered in a supplement? It has little biological relevance.

We agree with the reviewer, and have moved the “BBclass” annotation out of the main figure and into the supplemental heatmap figure (Figure S3).

Figure 3: This could be a lot clearer. A lot of information is expressed and it is hard to pick it apart. I would recommend multiple panels delivering discrete messages: 1) separate ACE2-RBD structure (i.e. without annotation) to orientate the reader (maybe a miniature inset of entire spike complex for context) 2) 4 example RBD-fAb structures illustrating the 4 clusters of antibodies (maybe context image of full spike in supplement to illustrate up/down conformation etc.) 3) structures annotating multiple antibodies (similar to what is already provided). Also, consistent color-coding of antibody clusters across figures is desirable. Clear communication in this figure will really aid the reader’s appreciation of the work.

Figure 3 has been modified as suggested by the reviewer, to include a separate ACE2-RBD representation (panel B), the clusters and ACE2 in the context of the spike (panel C), and the RBD with multiple representative antibodies bound (panel D).

Figure 4/5: The order of residues along the bottom is different to that in figure 2; is there a reason for this, can they be made consistent, again to ensure clarity?

The ordering of the columns in those figures was due to clustering of the heatmap data, i.e. hydrogen bonds or alanine ΔΔG values, thus was different than the ordering of the contact-based data in Figure 2. Though it is not straightforward to match the ordering exactly due to the different number of columns/positions in Figure 2 versus the other figures, we reoriented the dendrograms so that key positions highlighted in Figures 4 and 5 are in similar relative positions as in Figure 2.

Figure 6: this would be complemented with an RBD structure color-coded by conservation, alongside a structure annotated by antibody cluster for context.

We agree with this comment of the reviewer, and Figure 6 has been modified to add an RBD structure that is colored by conservation (panel B).

In my opinion, review and revision of the figures with a view on clarity of message will increase the impact of this work.

Minor Comments:

- *Line 96 states 8 angstrom cutoff whereas figure 1 legend states 7.*

The Figure 1 legend has been corrected to appropriately note 8 Å.

- *Line 119-120 needs rewording.*

Those lines have been reworded.

• Related to line 122 – I think more could be done to draw a relationship between the RMSD analysis (F1) and clustering (F2), might color coding help here?

We appreciate this suggestion from the reviewer, but we did not find a way to easily add the contact-based color codes from Figure 2 to Figure 1. However, we did added a reference to Figure 1 in the description of the clusters to more clearly link the results with regard to Cluster 1 (line 127).

• Line 127 – Figure S2 should be S3.

This has been corrected (now line 131) with the updated figure number (Figure S5).

• Line 175 – "lusters".
• Line 204, erroneous parentheses.
• Line 310-311, erroneous "and".

These typographical errors have been addressed in the manuscript (the text formerly at line 204 is no longer present due to revisions of the Results section).

Reviewer #3:
The authors performed structural and energetic analysis of SARS-CoV-2 S-RBD/antibody recognition after classifying antibodies by RBD binding residues using unsupervised clustering. Based on the structural data, they also evaluated the capacity of prevalent viral variant mutations to disrupt antibody recognition. Their study suggested that the cluster-2 antibodies were capable of targeting escape antibodies. The analysis should be able to serve as a useful reference for vaccine and therapeutic design. The computational work is solid and comprehensive though lacking experimental data to support some conclusions, e.g., cluster-2 antibodies are able to effectively neutralize escape SARS-CoV-2 variants while antibodies in other clusters are likely to be affected by these mutations. I recommend to publish this work after a few typos or minor errors are corrected.

We appreciate these comments from the reviewer.

1. Line 194-195, B.1.525 (E484K), and a recently reported variant of concern, B.1.526 (E484K). why B.1.525 and B.1.526 are the same? Do the authors mean L452R in B.1.526?

These lines regarding the variants have been revised, and the text noted by the reviewer is no longer present.

2. Line 311, delete the 'and' between identifies and antibody-RBD.

This typographical error was also noted by Reviewer 2 and has been corrected.
